# Sedentary behavior, cognition, and brain health in older adults: a systematic review

**DOI:** 10.3389/fnagi.2025.1622049

**Published:** 2025-07-08

**Authors:** Marissa A. Gogniat, Junyeon Won, Carlos Cruz, Amaya Aranda, Aryan Verma, Swathi Gujral, Andrea M. Weinstein, Afsara B. Zaheed, Keith R. Cole, Kelsie M. Full, Beth E. Snitz

**Affiliations:** ^1^Department of Neurology, University of Pittsburgh School of Medicine, Pittsburgh, PA, United States; ^2^Institute for Exercise and Environmental Medicine, Texas Health Presbyterian Hospital, Dallas, TX, United States; ^3^Department of Neurology, University of Texas Southwestern Medical Center, Dallas, TX, United States; ^4^Department of Bioengineering, University of Texas at Arlington, Arlington, TX, United States; ^5^Department of Psychiatry, University of Pittsburgh School of Medicine, Pittsburgh, PA, United States; ^6^Vanderbilt Memory and Alzheimer’s Center, Vanderbilt University Medical Center, Nashville, TN, United States; ^7^Department of Orthopaedic Surgery, Vanderbilt University Medical Center, Nashville, TN, United States; ^8^Division of Epidemiology, Department of Medicine, Vanderbilt University Medical Center, Nashville, TN, United States

**Keywords:** sedentary behavior, cognition, Alzheimer’s disease, brain health, older adult

## Abstract

Sedentary behavior has been associated with poor health outcomes, especially in older adulthood. Given that sedentary behavior is a highly prevalent, modifiable health behavior, there has been a recent increased interest in examining how sedentary behavior relates to cognition and brain health. The current body of literature is limited and mixed. The purpose of this systematic review was to examine the associations of sedentary behavior with cognition and brain health in older adults across the cognitive spectrum. This study was pre-registered with PROSPERO (CRD42023477868). Six comprehensive databases were searched with pre-registered search terms. A total of 33 studies were included. Overall, results indicated that greater sedentary behavior was associated with worse cognition and brain health, although associations varied based on differences in measurement and classification of sedentary behavior. We discuss next steps and implications for future research.

## 1 Introduction

Engaging in regular physical activity (PA) is a well-known behavioral strategy to maintain cognition and brain health (Won et al., 2021), and reduce risk for Alzheimer’s disease and related dementias (ADRD) (Iso-Markku et al., 2022). While a robust body of literature links participation in physical activity and exercise to improved cognition and brain health (Barella et al., 2010; Iso-Markku et al., 2018; Gogniat et al., 2022; Erickson et al., 2019), there has been significantly less research interest in sedentary behavior. Sedentary behavior is typically defined as any waking behavior characterized by an energy expenditure ≤1.5 metabolic equivalents (METs), while in a sitting, reclining or lying posture (Sedentary Behaviour Research Network, 2012). Sedentary behavior can also be operationalized into many different components including total time, breaks, and bouts amongst others (Tremblay et al., 2017). A distinct class of behaviors from physical activity and characterized by low energy expenditure (Biddle et al., 2004), sedentary behavior may be a lifestyle risk factor independently related to cognitive function and brain health in older adulthood. Greater sedentary time in older adulthood has been associated with several poor health outcomes including increased risk for cardiovascular disease, stroke, and all-cause mortality (Hajduk and Chaudhry, 2016; Wu et al., 2023). A prior systematic review in older adults aged 60 and older showed evidence from a small study (*N* = 649) that the average English older adult spends almost 9 h per day sedentary when measured objectively (Harvey et al., 2013). Evidence also shows that sitting for 12 h/day increased all-cause dementia risk by 63% (Raichlen et al., 2023). The high prevalence of sedentary behavior in older adulthood (Matthews et al., 2008) also lends importance to understanding the biological mechanisms by which it may accelerate risk for age-related cognitive decline and neurodegeneration.

Despite some evidence showing that sedentary time is associated with worse cognition in older adulthood, associations across studies are mixed. Results from various studies suggest that greater sedentary behavior is associated with worse global cognition (Wu et al., 2020), in addition to domain-specific associations, such as poorer executive function (Kesse-Guyot et al., 2012; Coelho et al., 2020) and memory (Bakrania et al., 2018). Other studies, however, show no impact of sedentary behavior on cognition (Yan et al., 2020; Maasakkers et al., 2020; Falck et al., 2017). Given the inconsistencies in study findings, there is a critical need to systematically examine the current literature to better understand the nature of these associations and what additional factors (e.g., measurement, study characteristics) may be driving these effects. Significant heterogeneity exists across studies in the measurement of sedentary behavior. Historically, sedentary behavior was measured via self-report, which may be faulty due to bias and unreliability in individuals with memory impairment (VandeBunte et al., 2022). The recent advent of wearable devices (e.g., wrist, thigh, hip) acquires more objective data, but presents some challenges including comparing across devices and setting appropriate activity cutpoints (Tremblay et al., 2017).

In addition to cognitive function, prior literature also supports the connection between sedentary behavior and brain structure and function in aging. For example, greater sedentary behavior has been associated with medial temporal lobe thinning (Siddarth et al., 2018), white matter atrophy (Arnardottir et al., 2016) and hyperintensities (Bronas et al., 2019), and lower cerebral blood flow (Zlatar et al., 2014). However, the pathophysiological mechanisms underlying these associations are poorly understood. Linking sedentary behavior to specific pathological brain changes would strengthen our understanding and inform prevention and intervention strategies aimed at improving brain health outcomes among older adults, which would be particularly important for those at risk for ADRD.

Given the recency of this literature base, there have been few systematic reviews in this area. A systematic review from Falck et al. (2017) included eight studies and concluded that sedentary behavior was associated with reduced cognitive function over the lifespan. Another systematic review on sedentary behavior and cognition with 13 studies was inconclusive (Olanrewaju et al., 2020). There have been far fewer reviews examining sedentary behavior and brain health. One prior review found a tentative association between habitual sedentary behavior and structural white matter (Maasakkers et al., 2022). Taken together, there is a need to provide updated information from a larger pool of studies that include updated sedentary behavior methodology, larger and more representative samples, and longitudinal follow-up to further understand these connections.

A PECO (Population, Exposure, Comparator, Outcome) framework (Morgan et al., 2018) was utilized to define the scope of this study. The purpose of this systematic review was to synthesize the current literature on associations between sedentary behavior (E) and cognition (O) AND sedentary behavior (E) and brain structure and function (O) in older adulthood (P) compared to those who do not engage in increased sedentary behavior (C) to provide a comprehensive understanding of associations that exist. We also examined the current literature in the context of different methodologies employed, outcomes measured, and risk of bias within studies. Overall, we hypothesized that greater sedentary behavior would be related to worse cognition and poor brain health. We also hypothesized that these relationships may vary based on the sedentary behavior mode of measurement (objective vs. subjective report) and the outcomes evaluated (e.g., comprehensive neuropsychological evaluation vs. cognitive screener; structural neuroimaging vs. functional neuroimaging).

## 2 Method

The current systematic review was conducted following the Preferred Reporting Items for Systematic Review and Meta-Analyses (PRISMA) guidelines (Page et al., 2021). This systematic review (CRD42023477868) was pre-registered on November 14, 2023 with PROSPERO International Prospective Register of Systematic Reviews and can be accessed at the following website: https://www.crd.york.ac.uk/PROSPERO/view/CRD42023477868.

### 2.1 Eligibility criteria

Inclusion criteria were the following: (1) peer-reviewed publications, (2) available in English, (3) cross-sectional and cohort/observational studies, (4) older adults ages 60 and older with or without cognitive impairment, (5) measurement of sedentary behavior at baseline, (6) objective cognition outcomes acquired by a validated assessment measure, (7) brain health outcomes measured via structural (volume, thickness, surface area, diffusion tensor imaging) and/or functional (fMRI, functional connectivity, cerebral blood flow) neuroimaging.

Exclusion criteria were the following: (1) study not available in English, (2) Intervention studies unless sedentary behavior and cognition/brain health are reported cross-sectionally at baseline, (3) studies that do not examine the associations of sedentary behavior with a cognition or brain health outcome, (4) participants younger than 60 years old, (5) participants with reported psychopathology or neurological disorders (e.g., depression, Parkinson’s Disease) other than Alzheimer’s disease.

### 2.2 Information sources

A search was conducted using EBSCOhost (MEDLINE, Academic Search Premier), Ovid (PsycINFO), ProQuest (PSYCArticles), PubMed, and Sedentary Behavior Research Database (SBRD) databases. The initial search began on December 8, 2023, and therefore all articles published prior to this date were eligible to be included in the search. Following this, reference lists from pertinent studies, reviews, and meta-analyses were manually searched by study authors (MAG, JW) for studies that may have not been captured in the original search. This search strategy was developed and pre-registered prior to beginning the search by MAG, JW, and SG.

### 2.3 Search strategy

An identical search strategy was applied to each database and included the following: (“older adults” OR “geriatrics” OR “aging” OR “seniors” OR “elderly” OR “healthy aging” OR “MCI” OR “dementia” OR “Alzheimer’s disease) AND (“sedentary behavior” OR “inactivity” OR “sitting” OR “low activity”) AND (“neuroimaging” OR “brain volume” OR “brain change” OR “MRI” OR “white matter” OR “connectivity” OR “PET” OR “cerebral blood flow” OR “cortical” OR “cognition” OR “memory” OR “thinking”). These search terms were developed by MAG, SG, and JW.

### 2.4 Study selection

Eligibility was assessed using the previously discussed criteria (see Eligibility Criteria). Following the initial search, study titles and abstracts were reviewed for preliminary determination of eligibility (i.e., sedentary behavior studies in older adults) by MAG. Studies that met the initial eligibility were then reviewed in detail to make a final decision on eligibility (MAG, JW, AA, CC). The corresponding authors were contacted when the data presented was insufficient to be able to determine final eligibility. When discrepancies assessment arose, MAG and JW discussed the studies until a consensus was reached.

### 2.5 Data collection process

A data extraction form was developed using Microsoft Excel and was consistent with the Cochrane Consumers and Communication Data Extraction Template (Ryan and Hill, 2019). Several authors (AA, CC, AV, JW, MAG) extracted the data, and discussions regarding data collection procedures (i.e., inclusion of demographic information, classification of outcomes) were routinely conducted via bi-weekly group meetings. All extracted data was verified by a second rater. The list of variables to be coded included the following: age, sex, education level, cognitive status (e.g., normal cognition, mild cognitive impairment, dementia), type of sedentary behavior (e.g., self-report, objectively measured, etc.), cognitive outcome (e.g., global cognition, memory, executive functions, processing speed, etc.), brain health outcome (e.g., volume, thickness, white matter integrity, functional connectivity, etc.), and bias in individual studies. For cohort studies where baseline sedentary behavior was related to cognition or brain health outcomes over time, follow-up time was extracted.

### 2.6 Risk of bias in individual studies

In order to assess risk of bias within individual studies, the NIH Quality Assessment Tool for Observational Cohort and Cross-Sectional Studies was used^1^, which uses 14 criteria to evaluate the methodological quality of cohort longitudinal and cross-sectional studies. Only one criterion: “Were the outcome assessors blinded to the exposure status of participants?” was not evaluated, as it was not applicable for observational studies with no status manipulation.

## 3 Results

### 3.1 Study selection

**Figure 1:**
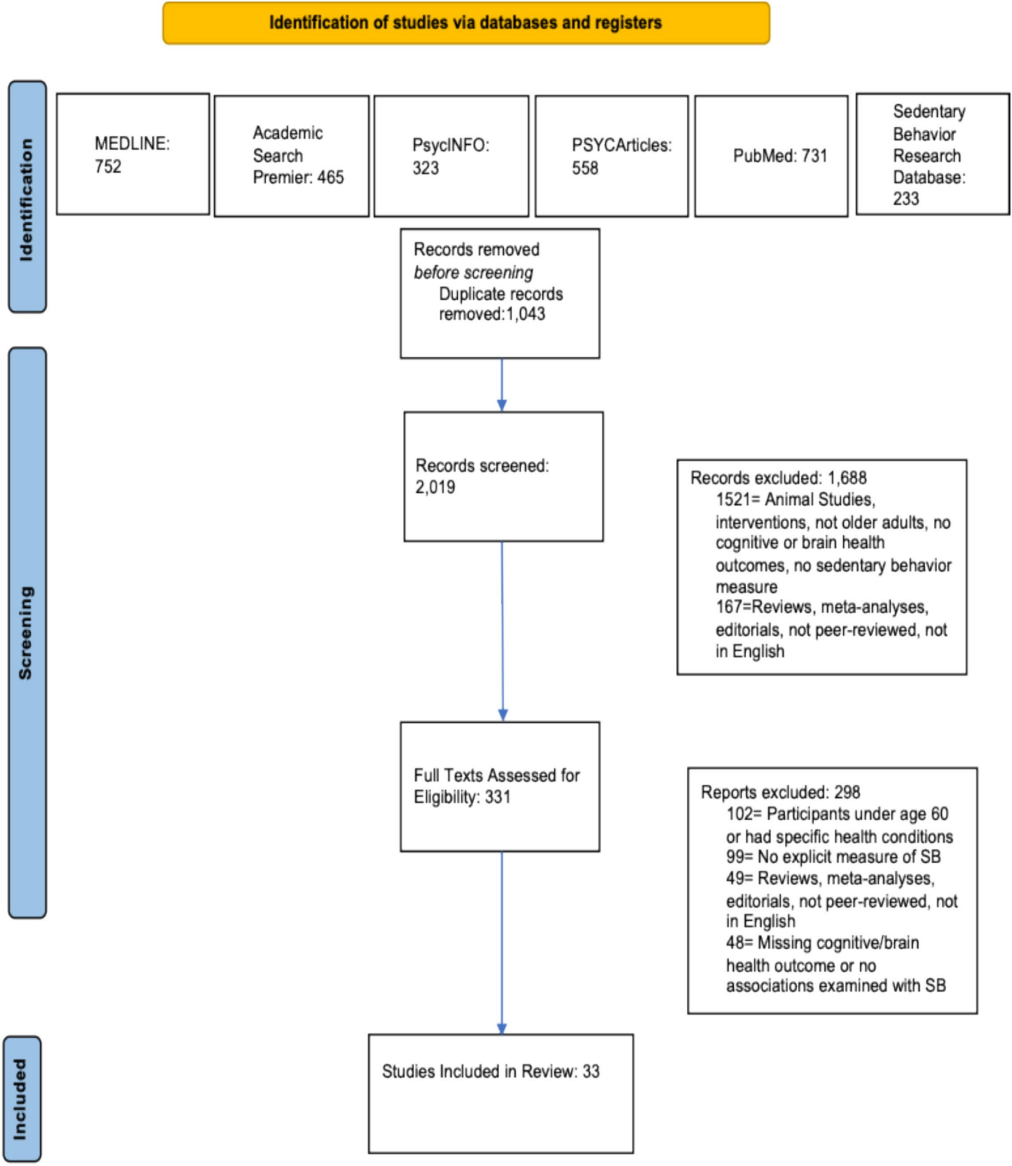
PRISMA flowchart. SB, Sedentary Behavior.

The initial search results from MEDLINE, PsycInfo, PsycArticles, Academic Search Premier, PubMed, and Sedentary Behavior Research Database returned 3,062 records. After removal of duplicates, 2,019 unique records remained. These 2,019 unique entries were initially screened using title and abstract review, and 1,688 records were excluded because based on this screen. Following this, 331 records were assessed for full eligibility using the criteria listed above (see Eligibility Criteria), and 298 records were excluded. No new records were included from the manual reference search. Final inclusion was 33 studies. This information is presented in a flowchart (see Figure 1) based on the PRISMA template (Page et al., 2021).

### 3.2 Study characteristics

Study characteristics for cognition (Table 1) and brain health (Table 2) were presented separately. Detailed study information including sample size, sample demographics (e.g., sex, age, education), cognitive status, and sedentary behavior are presented in Table 1 (Iso-Markku et al., 2018; Wu et al., 2020; Vance et al., 2005; Heisz et al., 2015; Steinberg et al., 2015; Rosenberg et al., 2016; Edwards and Loprinzi, 2017a; Edwards and Loprinzi, 2017b; Ku et al., 2017; Vásquez et al., 2017; Čukić et al., 2018; Wanigatunga et al., 2018; García-Hermoso et al., 2018; Folley et al., 2019; Zlatar et al., 2019; Amagasa et al., 2020; Burzynska et al., 2020; Suzuki et al., 2020; Maasakkers et al., 2020; Kurita et al., 2022; Chen et al., 2022; Gerten et al., 2022; Silva-Fernandes et al., 2022; Zhou et al., 2022; Shuai et al., 2023; Major et al., 2023; Han et al., 2023) and Table 2 (Arnardottir et al., 2016; Bronas et al., 2019; Zlatar et al., 2019; Burzynska et al., 2014; Engeroff et al., 2018; Dion et al., 2021; Machida et al., 2022). Two included studies had overlapping participants (Edwards and Loprinzi, 2017a; Edwards and Loprinzi, 2017b).

**Table 1:** Sedentary behavior and cognition.

Author (year)	*N* (Female %)	Mean age (SD)	Education	Cognitive status	SB assessment	Cognitive assessment	Summary of results
Vance et al. (2005)	158 (47%)	75 (6)	>HS	Unimpaired	Self-report	BVRT, TMT-B, Rey-O	SB ↑ and cognitive performance ↑
Heisz et al. (2015)	30 (50%)	74 (7)	>HS	Unimpaired	Self-report	Episodic (face) memory	SB ↑ and episodic memory ↓
Steinberg et al. (2015)	125 (66%)	77 (7)	>HS	Unimpaired	Self-report	CogState computerized battery	SB ↑ and executive function ↓
Rosenberg et al. (2016)	307 (72%)	84 (6)	>HS	NR	Accelerometer and Self-report	TMT A & B	Self-report SB ↑ and TMT-A performance ↑
Edwards and Loprinzi (2017a)	2,472 (55%)	70 (CI: 69–70)	NR	NR	Self-report	DSST	SB ↑ and DSST performance ↓
Edwards and Loprinzi (2017b)	2,472 (55%)	70 (CI: 69–70)	NR	NR	Self-report	DSST	SB ↓ and DSST performance ↑
Ku et al. (2017)*	274 (54%)	75 (6)	<HS	NR	Accelerometer	AD8	SB ↑ and AD8 performance ↓
Vásque et al. (2017)	1,496 (21%)	range = 65–75	>HS	NR	Accelerometer	B-SEVLT, WF, DSST	SB ≠ cognitive performance
Čukić et al. (2018)	1950’s: 310 (53%) 1930’s: 119 (55%) LBC1936: 271 (48%)	65 (1) 83 (1) 79 (0.4)	>HS	NR NR NR	Accelerometer	General cognitive ability, CRT, SRT	SB ≠ cognitive performance
Wanigatunga et al. (2018)	1,275 (67%)	79 (5)	>HS	Unimpaired and MCI	Accelerometer	Psychomotor speed, attention, working memory, memory	total SB ↑ and digit symbol coding performance ↓
Iso-Markku et al. (2018)	726 (52%)	73 (1)	<HS	NR	Accelerometer	TICS	SB ↑ and cognitive performance ↓
García-Hermoso et al. (2018)	989 (61%)	74 (7)	<HS	Unimpaired, MCI, dementia	Self-report	mMMSE	SB ↑ and global cognition ↓
Folley et al. (2019)*	8,475 (NR)	NR	NR	NR	Accelerometer	Pairs matching and Fluid Intelligence	SB ↑ and memory over time ↓
Zlatar et al. (2019)	52 (58%)	72 (5)	>HS	Unimpaired	Accelerometer	Executive function and memory composites	SB ≠ cognitive performance
Amagasa et al. (2020)	511 (53%)	73 (6)	<HS	Unimpaired and MCI	Accelerometer	MMSE (Japanese version)	SB ≠ cognitive performance
Burzynska et al. (2020)	228 (68%)	65 (5)	>HS	Unimpaired	Accelerometer	Virginia Cognitive Aging Project Battery	SB ↑ and vocabulary knowledge and reasoning ↑
Suzuki et al. (2020)	136 (50%)	88 (1)	<HS	Unimpaired, MCI, and dementia	Accelerometer	ACE-III	SB ↑ and global cognition in men ↓
Wu et al. (2020)	308 (57%)	69 (5)	<HS	Unimpaired and MCI	Accelerometer	MoCA	SB ≠ cognitive performance
Maasakkers et al. (2020)*	HELIAD: 1551 (60%) PATH: 1552 (49%) SALSA: 1663 (58%) SGS: 2597 (56%) SLAS2: 3087 (63%)	HELIAD: 73 (6) PATH: 75 (2) SALSA: 70 (7) SGS: 73 (6) SLAS2: 67 (8)	HELIAD: <HS PATH: > HS SALSA: < HS SGS: <HS SLAS2: <HS	Unimpaired and MCI	Self-report and Accelerometer	MMSE and 3MS	SB ↑ and global cognition in 1 study sample ↓ SB ≠ cognitive performance longitudinally
Kurita et al. (2022)	49 (46%)	78 (3)	>HS	Unimpaired and MCI	Self-report and Accelerometer	NCGG-FAT	SB (with cognitive activities) ↑ and cognition ↑
Chen et al. (2022)	1,681 (62%)	73 (6)	<HS	Unimpaired	Accelerometer	MoCA	SB (prolonged bouts) ↑ and MoCA orientation ↓
Gerten et al. (2022)	56 (54%)	76 (7)	>HS	Unimpaired	Accelerometer	Attention/psychomotor speed, executive function, memory	SB ↑ and verbal memory learning performance ↓
Silva-Fernandes et al. (2022)	32 (59%)	68 (4)	<HS	Unimpaired	Accelerometer	MoCA, language, executive function, processing speed, memory	SB ≠ cognitive performance
Zhou et al. (2022)	852 (60%)	80–84 (50% of sample)	<College	NR	Self-report	Immediate word recall	SB (type and total) ↑ and immediate word recall performance ↓
Shuai et al. (2023)*	5,356 (54%)	71 (7)	<HS	Unimpaired and MCI	Self-report	MMSE	SB (screen watching and cards) ↑ and MMSE ↓
Major et al. (2023)*	1,261 (52%)	75 (3)	>HS	Unimpaired	Self-report	3MS, DSST	SB (sitting time) ↑ and 3MS, DSST performance ↑
Han et al. (2023)	2019 (59%)	70 (5)	<HS	Unimpaired	Accelerometer	Memory, attention, verbal fluency, executive function, global cognition	SB ↑ and memory, verbal fluency ↓

*Denotes a longitudinal study, otherwise study is cross-sectional; ACE-III = Addenbrooke’s Cognitive Examination; AD8 = Ascertain Dementia 8-item Questionnaire; B-SEVLT = Brief Spanish English Verbal Learning Test; BVRT = Benton Visual Retention Test; CI = 95% Confidence Interval; CRT = Four Choice Reaction Time; DSST = Digit Symbol Substitution Test; HELIAD = Hellenic Longitudinal Investigation of Aging and Diet; HS = High School; LBC1936 = Lothian Birth Cohort 1936; MCI = Mild Cognitive Impairment; mMMSE = modified Mini Mental State Exam; MMSE = Mini Mental State Exam; MoCA = Montreal Cognitive Assessment; NCGG-FAT = National Center for Geriatrics and Gerontology-Functional Assessment Tool; NR = not reported; Rey-O = Rey-Osterreith Complex Figure Test; PATH = Personality and Total Health Through Life Project; SALSA = Sacramento Area Latino Study on Aging; SB = Sedentary Behavior; SD = standard deviation; SGS = Sasaguri Genkimon Study; SLAS2 = Singapore Longitudinal Ageing Study (II); SRT = Simple Reaction Time; TICS = Telephone Interview for Cognitive Status; TMT = Trail Making Test; WF = Word Fluency Test; 3MS = Teng Mini-Mental State Examination.

**Table 2:** Sedentary behavior and brain health.

Author (year)	*N* (Female %)	Mean age	Education	Cognitive status	SB assessment	Brain health outcome	Results
Burzynska et al. (2014)	88 (66%)	65 (4)	>HS	Unimpaired	Accelerometer	WMH volume and DTI	SB ↑ and parahippocampal WM FA ↓
Arnardottir et al. (2016)*	352 (61%)	Men: 79 (4) Women: 79 (5)	NR	Unimpaired	Accelerometer	GM, WM, WMH,	SB ↑ and WM volume ↓ at 5-yr follow-up
Engeroff et al. (2018)	50 (NR)	75 (7)	>HS	Unimpaired	Accelerometer	Hippocampal volume measured via MRS	SB ≠ hippocampal volume
Bronas et al. (2019)	94 (51%)	68 (7)	>HS	Unimpaired	Self-report	WMH volume	SB ↑ and WMH volume ↑
Zlatar et al. (2019)	52 (58%)	72 (5)	>HS	Unimpaired	Accelerometer	CBF	SB ↑ medial and lateral frontal regions CBF ↓
Dion et al. (2021)	18 (50%)	67 (6)	>HS	Unimpaired and MCI	Accelerometer	Functional connectivity	SB ↑ and CEN connectivity ↓
Machida et al. (2022)	485 (53%)	73 (6)	<HS	NR	Accelerometer	Hippocampal volume	SB ≠ hippocampal volume

*Denotes a longitudinal study, otherwise study is cross-sectional; CBF = cerebral blood flow; CEN = central executive network; DTI=Diffusion Tensor Imaging; FA = fractional anisotropy; GM = gray matter; HS=High School; MRS = magnetic resonance spectroscopy; NR = not reported; SB=Sedentary Behavior; SD = standard deviation; WM = white matter; WMH = white matter hyperintensities.

The 33 included studies resulted in a total of 43,577 participants (*M* = 1,321, SD = 2,375, range = 18–10,450). The sample displayed some variability in age (*M* = 73, SD = 5, range = 65–88) and was, on average, gender balanced (% female; *M* = 56%, SD = 9%, range 21–72%). Of the 28 studies that reported education level, 57% reported a majority completing high school or higher, while 43% reported a majority of the sample completing less than high school. Only 23 studies explicitly reported on the cognitive status of the sample. Most studies utilized cognitively healthy samples (*n* = 14), and nine studies included participants with mild cognitive impairment (MCI) and/or dementia.

Regarding measurement of sedentary behavior, a majority of studies (*n* = 20) used purely objective measures, followed by subjective measures (*n* = 10), and then combination of both objective and subjective methods (*n* = 3). Of the studies that used an objective measure of sedentary behavior and reported the device location, the majority of studies used a hip-worn device placement (*n* = 16), with one study using a wrist-worn device and one study using a thigh-worn device (*n* = 5 did not put wear location but hip is suspected based on device type). The most common devices utilized were versions of the Actigraph (GT3X, GT3X+, GTM1; *n* = 14), and the majority of studies focused on total sedentary time using <100 counts per minute (*n* = 13). In studies that utilized a subjective measure of sedentary behavior (*n* = 13), all studies included participant self-report of sedentary behavior. Of these 13 studies that utilized some sort of self-report, some (*n* = 6) used a validated measure with several questions (e.g., Sedentary Behavior Questionnaire (Rosenberg et al., 2010)), while the rest utilized 1–3 individual questions (*n* = 7).

Among studies that reported a cognitive outcome (*n* = 27), the most commonly used assessment was a single-domain cognitive measure or composite scores (e.g., executive function composite; *n* = 11), followed by a cognitive screening tool (e.g., Mini Mental Status Exam; [MMSE]) (*n* = 10), and finally, fewer studies included more comprehensive neuropsychological batteries (*n* = 6). A majority of the studies with cognitive outcomes (*n* = 15/27) reported a negative association between sedentary behavior and cognitive performance, indicating that greater sedentary time was associated with worse cognitive performance. Five studies reported positive associations between sedentary behavior and cognitive performance, and seven studies reported no or mixed associations between sedentary behavior and cognitive performance.

Of the studies that reported brain structure and function outcomes (*n* = 7), the most common outcome measured was white matter hyperintensities (WMH; *n* = 3), followed by hippocampal volume (*n* = 2), and functional neuroimaging outcomes (*n* = 2). All brain imaging studies reported scanner strength: most studies utilized a 3 T scanner (*n* = 5) while two studies utilized a 1.5 T scanner. Five of the brain health studies reported negative associations between sedentary behavior and measures of brain health, while two studies reported no association between sedentary behavior and hippocampal volume.

Most studies were cross-sectional (*n* = 27/33) with only 1/6 longitudinal studies belonging to the brain health category. A majority of the longitudinal studies (*n* = 5/6) reported negative associations between sedentary behavior and cognitive/brain health outcomes. The mean follow-up time for longitudinal studies was 3.6 years. One study (Zlatar et al., 2019) contained both cognitive and brain health outcomes, and those results are presented separately in both tables. Two studies with cognitive outcomes utilized participants from the same sample (Edwards and Loprinzi, 2017a; Edwards and Loprinzi, 2017b).

### 3.3 Risk of bias results

**Figure 2:**
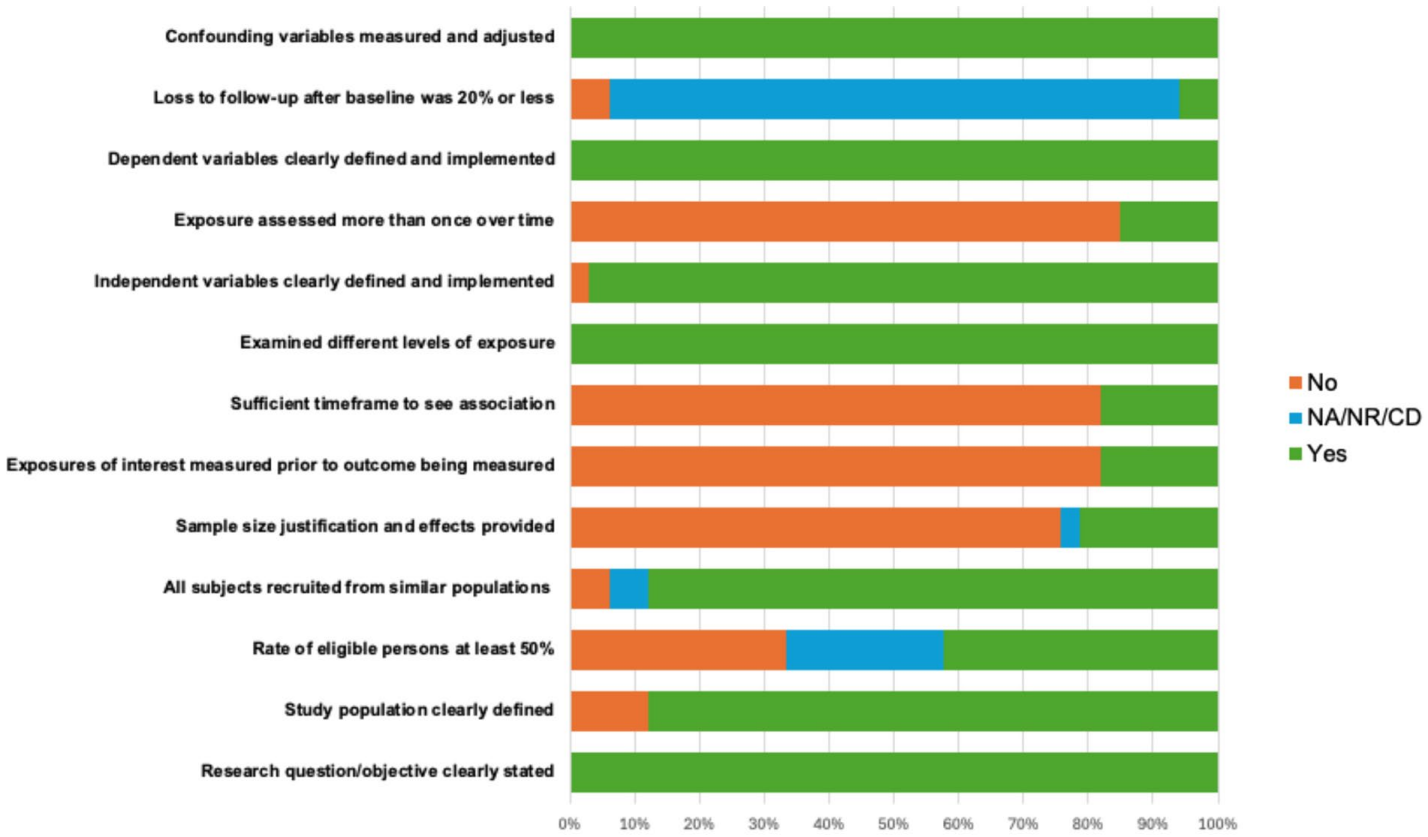
Risk of bias plot. NA = Not Applicable; NR = Not Reported, CD = Cannot Determine.

The risk of bias (ROB) assessment revealed consistent, reasonable methodological standards for the majority of studies, with 73% of the studies rated overall as “good” and 27% of the studies scoring “fair” taking into account their methodological rigor. Nearly all studies included clear statement of the research objective (100%), defined the study population (88%), recruited subjects from similar populations (88%), defined independent (97%) and dependent variables (100%), examined different levels of exposure of the independent variable (100%), and adequately addressed confounding variables (100%). Across all studies, lower endorsed ROB categories included providing sample size justification (21%) and obtaining participation rate above 50% of the total eligible sample (42%). In addition, due to most of the studies being cross-sectional, time-dependent categories such as assessing exposure more than once (15%) or providing sufficient time frame to observe associations (18%) were less frequent. ROB metrics are depicted in detail in Figure 2.

### 3.4 Synthesis of results

### 3.5 Sedentary behavior and cognition

As mentioned above, most of the studies in this systematic review contained cognitive outcomes (vs brain health outcomes). The majority of studies indicated that there was a negative association between sedentary behavior and cognition (Iso-Markku et al., 2018; Heisz et al., 2015; Steinberg et al., 2015; Edwards and Loprinzi, 2017a; Edwards and Loprinzi, 2017b; Ku et al., 2017; Wanigatunga et al., 2018; García- Hermoso et al., 2018; Folley et al., 2019; Suzuki et al., 2020; Chen et al., 2022; Gerten et al., 2022; Zhou et al., 2022; Shuai et al., 2023; Han et al., 2023), while the rest of the studies reported mixed or no effect (Wu et al., 2020; Vásquez et al., 2017; Čukić et al., 2018; Zlatar et al., 2019; Amagasa et al., 2020; Maasakkers et al., 2020; Silva-Fernandes et al., 2022), or positive effects (Vance et al., 2005; Rosenberg et al., 2016; Burzynska et al., 2020; Kurita et al., 2022; Major et al., 2023). A majority of the studies assessed cognition using a cognitive screening tool or a more limited cognitive battery. Of the five studies reporting positive associations between sedentary behavior and cognition, two studies utilized self-report of sedentary behavior, two utilized self-report and objective measurement, and only one used objective measurement. Five studies were longitudinal (Ku et al., 2017; Folley et al., 2019; Maasakkers et al., 2020; Shuai et al., 2023; Major et al., 2023). Of these five, most found a negative association between sedentary behavior and cognition (3/5), while 1 study found no association, and 1 found a positive association (see Table 1).

### 3.6 Sedentary behavior and brain health

Seven studies investigated the associations of sedentary behavior with brain structure and function measured via magnetic resonance imaging (MRI) and magnetic resonance spectroscopy (MRS). Specifically, observational studies reported that greater sedentary behavior was associated with increases in WM damage (Bronas et al., 2019), deterioration of microstructure organization (Burzynska et al., 2014), decrease in regional CBF (Zlatar et al., 2019), and reduction in functional network connectivity (Dion et al., 2021) in older adult samples. Furthermore, a longitudinal study found that high sedentary behavior at baseline was associated with reduction in WM volume at 5-year follow-up (Arnardottir et al., 2016). In contrast, no associations of sedentary behavior with hippocampal volume were observed. In terms of sedentary behavior measurements, all the studies used objective measurement of sedentary behavior using accelerometry, except one study (Bronas et al., 2019), which used self-reported measurement of sedentary behavior (see Table 2).

## 4 Discussion

### 4.1 Measurement of sedentary behavior

Our systematic review of the literature suggests that accelerometry is the most popular method of sedentary behavior measurement. The majority of the studies, especially in the most recent studies, assessed the associations of sedentary behavior with cognition (*n* = 15) utilizing an accelerometer. This is likely because accelerometry technology and validation for use of sedentary behavior in older adulthood has improved (Heesch et al., 2018), and this methodology is becoming more easy to implement. A minority of studies utilized self-report measures only (*n* = 9), mostly with validated questionnaires, or combined self-report and accelerometry methods (*n* = 3). Surprisingly, almost all studies except for one that examined brain health outcomes utilized an accelerometer. It is important to note that self-report has some important considerations, as older adults, particularly with cognitive impairment, pay not be as accurate in their reporting (VandeBunte et al., 2022).

Studies that used accelerometry varied in the devices used, the placement of devices, and the data processing cutpoints applied to classify sedentary level cut-offs, which has well-documented implications for the accuracy of capturing activity level, particularly in older adults (Schrack et al., 2016), and using wrist-worn devices (Wu et al., 2020). Compared to placing the accelerometer device on the wrist or hip, thigh device placement is considered the gold standard for the measurement of sedentary behavior because it captures positional information and postural changes (Kozey-Keadle et al., 2011). Thigh-worn devices were only utilized in one study reviewed in this investigation (Čukić et al., 2018). Future research should consider how device type, processing methodology, and placement in interpretation of findings. In addition, as physical activity and sedentary behavior are interrelated but separate behaviors (Dogra and Stathokostas, 2012), study results may differ based on whether physical activity level was adjusted for in analyses. As the field evolves in identifying best practices, future work might consider the importance of developing standardization procedures.

### 4.2 Classification of sedentary behavior

Many studies with self-report of sedentary behavior differed in the way sedentary behavior was categorized, and this could be a potential explanation for why results were not always consistent. Studies that examined specific domains of sedentary behavior may be better equipped to disentangle these discrepancies, as cognitively stimulating sedentary activities may be less detrimental or even helpful to cognition. For example, Major et al. (2023). found that higher amounts of sedentary reading time was positively associated with cognition, while participants who increased their TV watching time in particular, had significantly lower global cognition. Shuai et al. (2023) found that longer screen watching and playing cards was related to better global cognition, while other forms of sedentary behavior that did not involve screen time or playing games were associated with worse global cognition. In addition, not only did the category of sedentary behavior appear to sometimes be differentially related to cognition, but also perhaps the type as well. For example, Chen et al. (2022) found that sedentary time that accumulated in prolonged bouts, but not total sedentary time, was inversely associated with cognitive orientation ability among older adults. These examples demonstrate the importance of specifying the type of sedentary behavior as another important methodological consideration.

## 5 Conclusion and future directions

**Figure 3:**
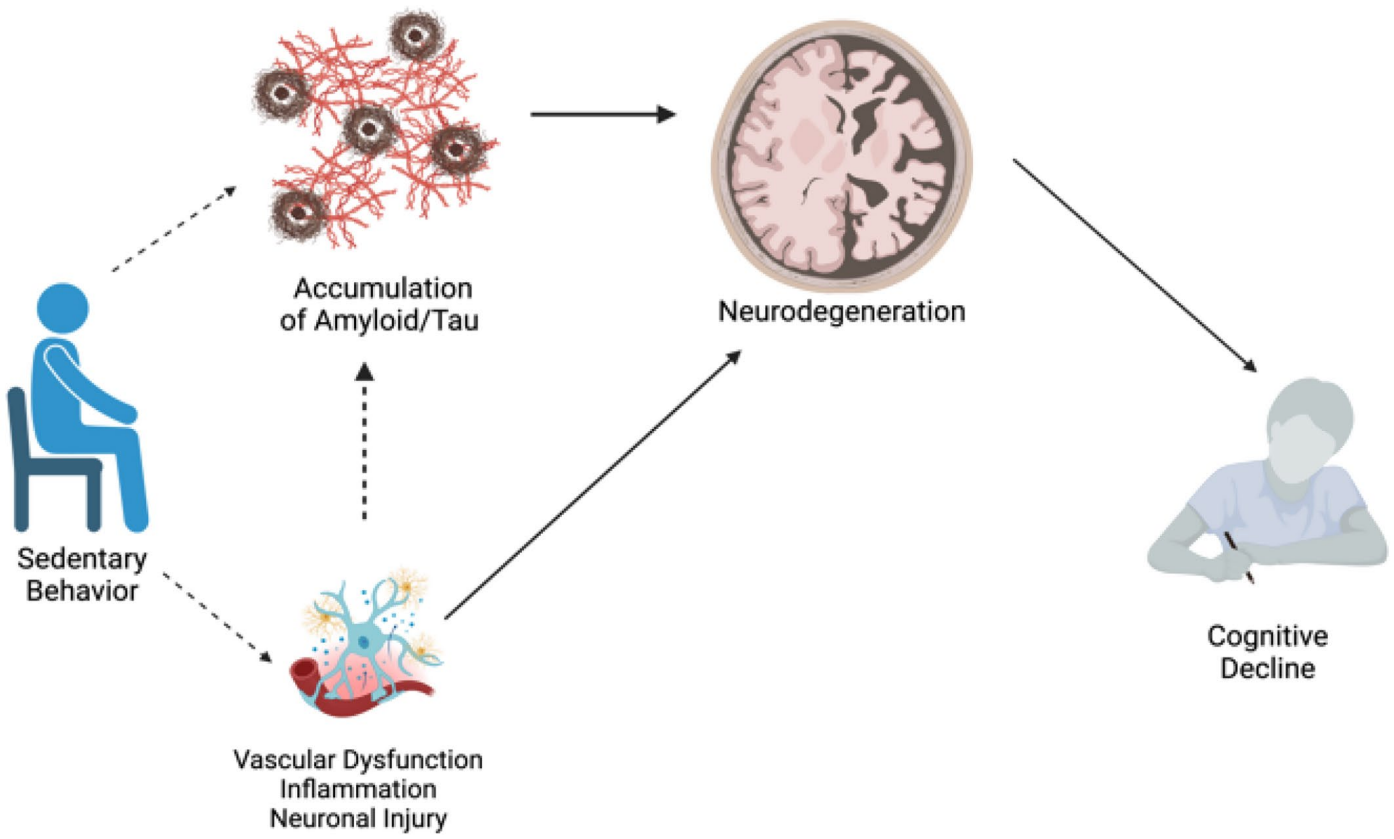
Conceptual diagram: potential pathways connecting sedentary behavior to AD risk. Dotted lines represent pathways to be tested, solid lines represent pathways with strong literature support.

We systematically searched and reviewed 33 studies, and these studies generally had a low RoB. Like all systematic reviews, there were limitations to our search including the inability to include all possibly relevant databases, broad search terms, ability of the study team to only include studies that were written/translated into English, and the possibility of publication bias in included studies. However, the results of this systematic review suggest that overall, greater sedentary behavior is negatively associated with cognition and with brain health (see Figure 3 for a conceptual summary diagram). Researchers should consider several methodological factors including how sedentary behavior was measured (objective vs. subjective) and classified (e.g., sedentary time watching TV, working). Additional research is needed to better understand how the type and/or quantification of sedentary behavior (e.g., cognitively stimulating vs. not; total time vs. bouts vs. duration) influences its associations with cognition and brain health, as this may have an impact on the directionality of results (Wanders et al., 2021). Few studies in this review utilized both self-report and accelerometer measurement of sedentary behavior, although this may not be feasible or reliable in an older adult population with cognitive impairment. In addition, few studies had longitudinal measures of cognitive and brain health outcomes, limiting our ability to understand the direction of these associations over time. Given that there is evidence that the negative effects of sedentary behavior are likely cumulative (Diaz et al., 2017) and may be like other lifestyle factors in midlife that are critical for cognitive aging (Barnes and Yaffe, 2011; Livingston et al., 2024), more longitudinal research is needed to determine directionality and draw stronger conclusions. Finally, we sought to examine these associations across the cognitive spectrum from healthy aging to ADRD. Unfortunately, few studies included participants with MCI, and even fewer with dementia. Future work should consider including participants with a wider range of cognitive abilities to assess whether these associations may differ based on cognitive status.

## Data Availability

The original contributions presented in the study are included in the article/supplementary material, further inquiries can be directed to the corresponding author/s.

## Footnotes

^1^ https://www.nhlbi.nih.gov/health-topics/study-quality-assessment-tools
